# Utilization of a State Run Public Private Emergency Transportation Service Exclusively for Childbirth: The Janani (Maternal) Express Program in Madhya Pradesh, India

**DOI:** 10.1371/journal.pone.0096287

**Published:** 2014-05-14

**Authors:** Kristi Sidney, Kayleigh Ryan, Vishal Diwan, Ayesha De Costa

**Affiliations:** 1 Public Health Sciences, Karolinska Institutet, Stockholm, Sweden; 2 Public Health and Environment, R.D. Gardi Medical College, Ujjain, Madhya Pradesh, India; 3 International Center for Health Research, R.D. Gardi Medical College, Ujjain, Madhya Pradesh, India; Aga Khan University, Pakistan

## Abstract

**Background:**

In 2009 the state government of Madhya Pradesh, India launched an emergency obstetric transportation service, Janani Express Yojana (JEY), to support the cash transfer program that promotes institutional delivery. JEY, a large scale public private partnership, lowers geographical access barriers to facility based care. The state contracts and pays private agencies to provide emergency transportation at no cost to the user. The objective was to study (a) the utilization of JEY among women delivering in health facilities, (b) factors associated with usage, (c) the timeliness of the service.

**Methods:**

A cross sectional facility based study was conducted in facilities that carried out > ten deliveries a month. Researchers who spent five days in each facility administered a questionnaire to all women who gave birth there to elicit socio-demographic characteristics and transport related details.

**Results:**

35% of women utilised JEY to reach a facility, however utilization varied between study districts. Uptake was highest among women from rural areas (44%), scheduled tribes (55%), and poorly educated women (40%). Living in rural areas and belonging to scheduled tribes were significant predictors for JEY usage. Almost 1/3 of JEY users (n = 104) experienced a transport related delay.

**Discussion:**

The JEY service model complements the cash transfer program by providing transport to a facility to give birth. A study of the distribution of utilization in population subgroups suggests the intervention was successful in reaching the most vulnerable population, promoting equity in access. While 1/3 of women utilized the service and it saved them money; 30% experienced significant transport related delays in reaching a facility, which is comparable to women using public transportation. Further research is needed to understand why utilization is low, to explore if there is a need for service expansion at the community level and to improve the overall time efficiency of JEY.

## Introduction

In order to improve population coverage of maternal health care, reduce inequity in access and move towards the achievement of Millennium Development Goal (MDG) 5 (reduction in maternal mortality); governments in South Asia have implemented innovative demand side financing initiatives over the last decade [1]. The most well-known of these is India’s large cash transfer program, the Janani Suraksha Yojana (JSY), to reduce maternal mortality by promoting in-facility delivery [2].

Though not often explicitly stated, the main argument cited in favor of using demand side financing programs, like the JSY, is that beneficiaries often experience financial barriers that prevent them from using a particular service or intervention, in this case institutional delivery. The financial barrier argument also applies to overcoming physical access barriers i.e. geographical distance. Therefore providing either transport or funds designated for that purpose is seen as a way of overcoming barriers such as the absence of a reliable public transport system, difficulty in organizing transportation at short notice, and the extremely high out-of-pocket cost of organizing such transport [3]. By addressing geographical barriers, the transport subsidy/service acts in synergy with the JSY cash transfer program to increase utilization of facilities for institutional birth. While there has been considerable focus on the cash transfer to reduce financial access barriers to institutional delivery, little attention has been paid to efforts directed at simultaneous reduction of geographic access barriers [4], [5].

The JSY program originally allocated a separate smaller payment (USD 5) to compensate beneficiaries for travel expenses [6]. In 2008, despite these payments, institutional delivery remained below 50% in Madhya Pradesh, a large central Indian province with high a maternal mortality [7]. Organizing and paying for emergency transport privately was still likely a deterrent and negatively influenced the decision to travel to a facility for delivery. The state department of health in conjunction with UNICEF chose to make emergency obstetric transportation available under a unique public private initiative, the Janani Express Yojana (JEY or maternal express program). The government contracted private agencies to provide transportation free of charge to pregnant women so they could deliver in a health facility [6], [8].

In 2006, the state government piloted JEY and implemented the service state-wide in 2009. Since its inception, the health department in Madhya Pradesh reports that over 300,000 women have used the JEY transport to reach a hospital for delivery [8]. However, there have been few academic reports studying the utilization and equity in access of JEY in transporting mothers, particularly poor mothers most at risk of morbidity/mortality. The objectives of this paper are to study (a) the utilization of JEY among mothers delivering in public health facilities (i.e. JSY beneficiaries) and private facilities, (b) the characteristics and predictors of use, and (c) the delays experienced by women who used the JEY transport compared to women who used alternative modes of transportation. This paper is relevant considering the importance of emergency obstetric transport in the context of achieving MDG5 and the fact that many low-middle income countries are looking for innovative solutions to make emergency transport more readily available. The specific experience in Madhya Pradesh with this unique public private initiative is relevant to many other low and middle income settings.

## Methods

Ethics Statement: Ethical approval was granted by the Ethics Committee of R.D. Gardi Medical College, Ujjain. Written informed consent was given by all study participants.

### Study Setting

Madhya Pradesh state is located in the central part of India; 77% of its 72 million population live in rural areas, 31% live below the poverty line (BPL) [9]. It has relatively poor health indicators; infant mortality ratio stands at 65 per 1 000 live births and maternal mortality ratio (MMR) is 277 per 100 000 live births [10]. The female literacy rate ranges from 31% to 76%. The state is divided into 51 administrative districts, each with a population of 1–2 million [11]. Each district has its own district health administration that is responsible for the implementation of the JEY, the finalization of contracts with local transport operators under the JEY and oversight of the JEY. Our study was conducted in three purposively selected districts of Madhya Pradesh. These districts were selected to reflect different levels of socioeconomic development, MMR, institutional delivery uptakes, and geographic areas. The characteristics of the three districts are depicted in Table 1.

**Table 1:** Background characteristics of the three study districts, Madhya Pradesh (MP).

	District 1	District 2	District 3	MP
**Total Population** (million)[11]	2	1	1.1	72
**Rural Population (%)** [11]	58	88	71	68
**Female literacy (%)** [11]	61	56	58	60
**Human Development Index (HDI)** [27]	0.6	0.5	0.6	0.4
**Crude Birth Rate** [10]	24	31.5	24.2	24.8
**Institutional delivery (%)** [10]	81	72	58	76
**MMR** [10]	206	386	415	277
**Number of JEY vehicles per district** [12]	18	24	18	168

#### Structure of the health system

The health infrastructure in the study area is similar to the overall health system in India; a mix of public and private providers. In each district, the public sector in rural areas consists of a three-tier structure; (i) at the lowest level, a sub-center run by a female health worker; (ii) at the intermediary level, a Primary Health Center (PHC) with a medical officer and other paramedical staff; and (iii) at the higher level, a Community Health Center (CHC) with obstetric specialists and inpatient beds. Tertiary care is provided by the district hospital located in each district headquarter town. The public sector is the dominant provider of facility based delivery services in Madhya Pradesh. The private sector contributes to a very small proportion of obstetric services in the state and is concentrated predominantly in urban areas.

#### The janani (maternal) express yojana

**Figure 1:**
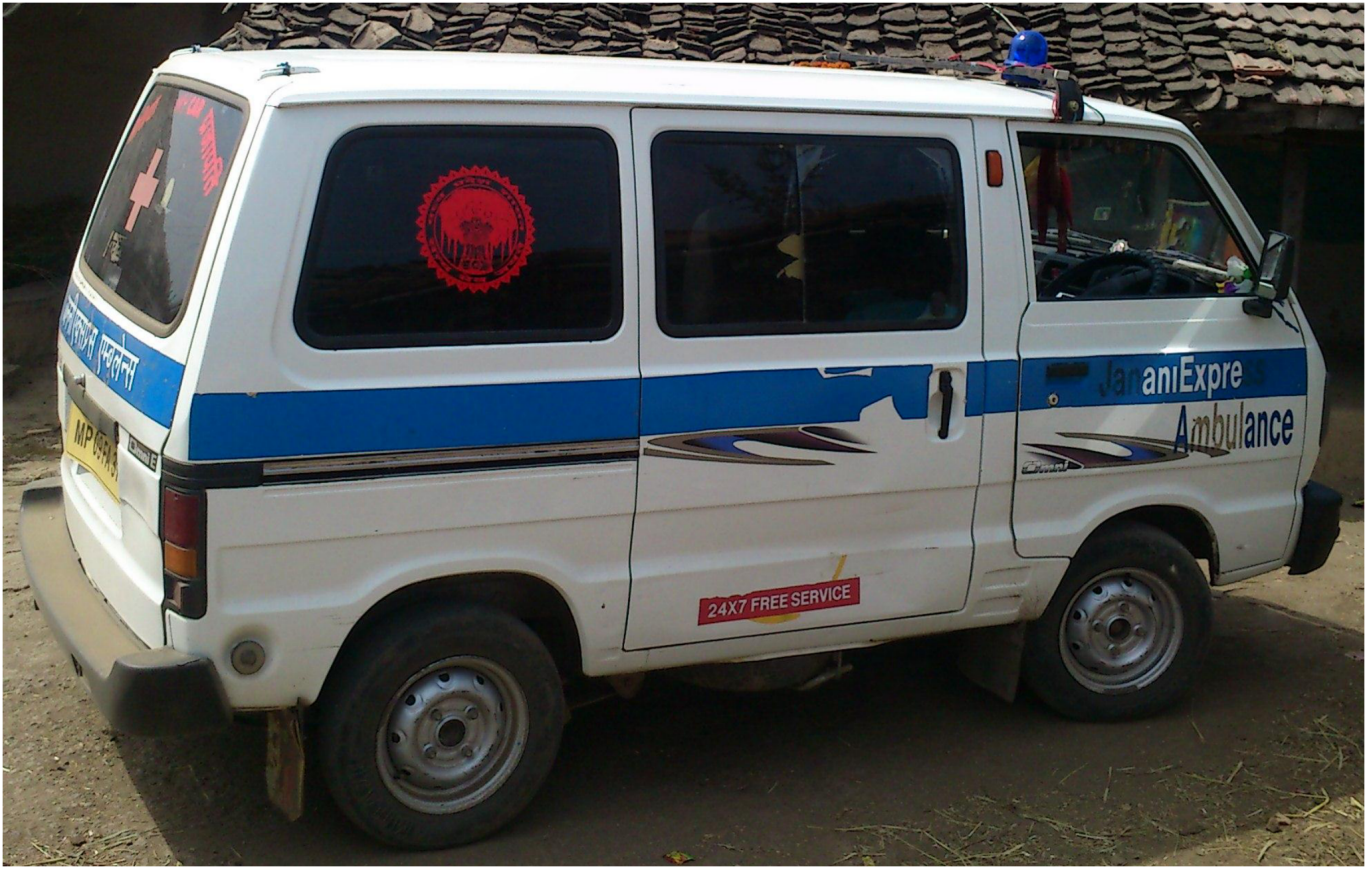
Janani Express Yojana Vehicle.

The JEY comprises a fleet of vans (depicted in figure 1) stationed at each public health facility. Currently, 893 vehicles operate in the 51 districts under JEY [12]. Contracts to run the JEY service are tendered by the department of health at the district level. Different local private transport agencies operating in the district respond to these tenders. The government stipulates the type of vehicle and the basic requirements that should be included; a stretcher, water, curtains to provide privacy, a basic first aid kit, and a disposable delivery kit in case of an emergency delivery on the way.

#### Responsibilities of the private agency

The private agency is responsible for procuring the vehicle, on-going maintenance and upkeep of the vehicle, and transporting mothers from their homes to the nearest hospital [8]. The state department pays the contracted private agency a fixed amount up to rupees (Rs.) 20,000 ($325) per month for the first 1500 kilometers (km); Rs. 5 ($0.08) is received for any additional km traveled.

#### Centralized call center model

**Figure 2:**
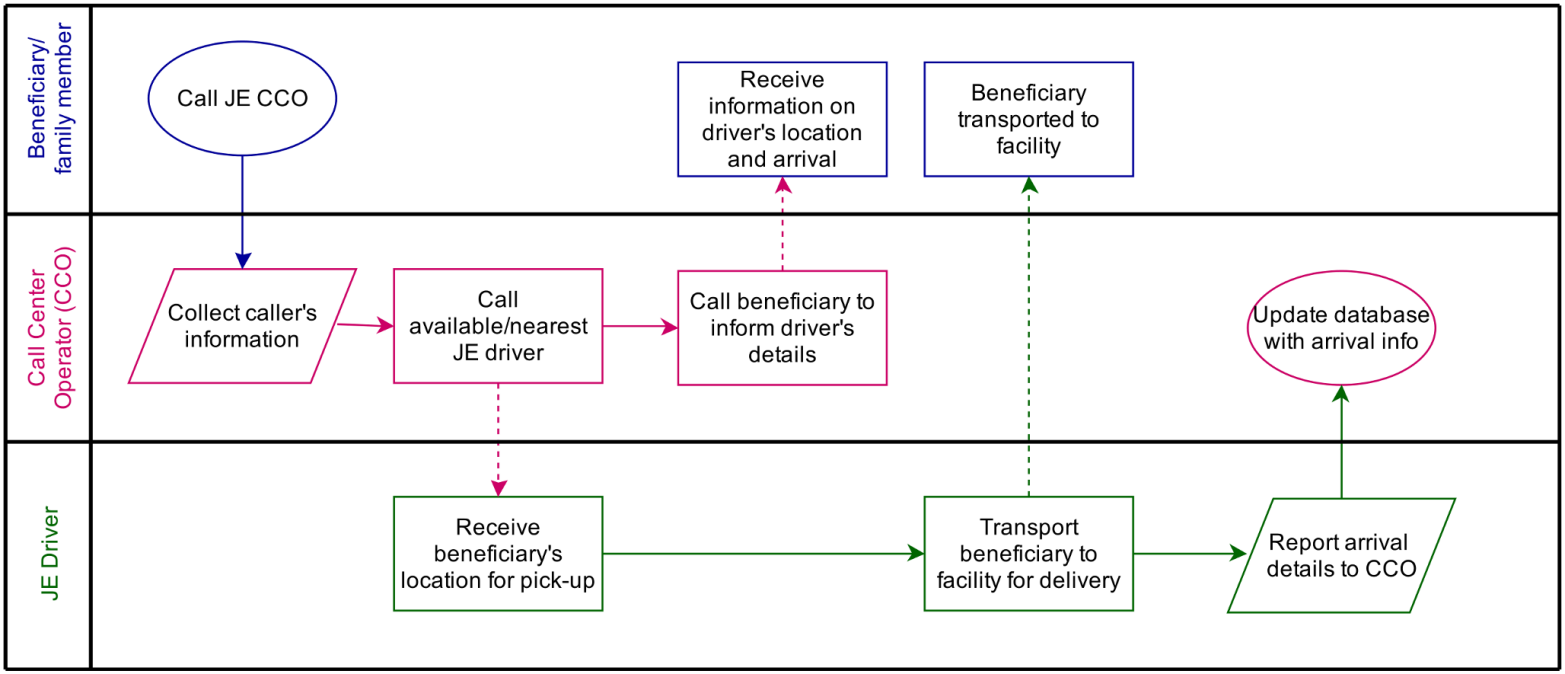
Flow chart depicting the JEY vehicle dispatching process.

Each district operates a separate call center (supported by UNICEF), functional 24 hours a day, where a pregnant woman or her family members can call to requisition a JEY vehicle when needed. Once a call is received, the call center operator collects personal details and information on the location and a JEY vehicle is dispatched. The woman is then transported to the nearest public facility. The overall process is shown in figure 2. JEY vehicles can also be used for inter-facility referrals.

#### The JEY in the context of the JSY

JSY cash transfer program is operational throughout India, but eligibility criteria differ depending on the state. In Madhya Pradesh, all women who deliver in public sector institutions (primary health care facilities, community health centers, and the district hospital) are eligible for the cash transfer regardless of their poverty status. Since 2004, the institutional delivery proportion has increased substantially from 28% to 76% in 2012 [10], [13]. Although the actual magnitude of JEY’s impact on this increase is unclear, the service functions within the context of JSY and supports the efforts of increasing institutional delivery.

### Study Design

A cross-sectional study performed in health facilities.

### Data Collection

An initial list of all health facilities (n = 931) and the number of deliveries performed in these facilities during 2011 was obtained from the state government for each of the three study districts. Facilities conducting less than 10 deliveries (n = 832) in a month were excluded and three facilities declined to participate. All facilities that conducted more than 10 deliveries a month (n = 96) were surveyed by a trained research assistant between February 2012 and April 2013. These 96 facilities accounted for 97% of all institutional deliveries in 2011 for the three study districts. Details on the selection of facilities are shown in appendix S1.

The research assistant who visited each study facility recruited all women who delivered in the facility during a five day consecutive period. Women were interviewed to elicit information on their basic socio-demographic characteristics, details of travel to the facility, residence prior to delivery, and if the accredited social health activist (ASHA) accompanied her to the facility. The ASHA is a female resident of the village who is incentivized by the government to motivate women to deliver at facilities under JSY [28]. They were also asked reasons for delay if a period of more than two hours was reported between deciding to leave their home and arriving at the facility.

During this period, RAs also obtained information on emergency obstetric care (EmOC) signal functions performed in the last three months at each facility. Facilities were classified based on the performance of the six signal functions [14] as providing EmOC services or not.

### Variables

#### Dependent variables

1. *Janani Express Yojana (JEY) User*: JEY user was considered to be any mother that reported arriving at the health facility exclusively by the JEY transport vehicle. 2. *Transport Delay*: A transport delay was considered to have occurred when the women reported a time of greater than 120****minutes from deciding to leave home and arriving at the health facility and the reason reported was related to transport (waiting for, organizing, or finding transportation). A delay was of more than 120****minutes was selected as the cut-off because (i) WHO and UNFPA recommend women should have access to EmOC facilities within 120****minutes [14] and (ii) it is reported to be significantly associated with in-hospital maternal mortality [15].

#### Independent variables

*Education*: Education was coded into two groups: low educational status (no education or only primary education) and high educational status (any higher level than primary). *Below the poverty line (BPL)*: BPL status was obtained by asking women if they/their family possessed a BPL card. *Caste*: Caste/tribe reported by the mother was categorized into general caste, scheduled caste, other backward castes, and scheduled tribe, as classified by a government list. *Place of residence*: Place of residence reported by the mothers was recorded as rural/urban based on the Census of India definition. *Distance Travelled*: The nearest road route distance between the residence of the woman just before delivery and location of the facility was calculated in km. This was obtained using network analysis tool of the program ArcInfo. *ASHA accompanied*: Women were asked if an ASHA had accompanied them to the facility. *Referred Mothers*: Women who reported being sent in from another health facility for further care.

#### Variable definitions

The BPL card is issued by the Government of India to indicate financially disadvantaged individuals or households. The remuneration is based on various parameters including land ownership, type of house, sanitation, food security, household goods, literacy status, means of livelihood, etc. Card holders benefit from welfare programs [29]. Scheduled castes, backward castes and scheduled tribes were groups of people historically subject to social disadvantage and exclusion for different reasons. They were awarded special status by the Constitution of India under a national positive affirmation policy that entitles them to specific social benefits [30].

### Data Management

Data was managed using research electronic data capture (REDCap). REDCap is a secure web-based application designed to support data capture for research [16].

### Statistical Methods

Data was analyzed in STATA version 12. Univariate analyses were described using median and interquartile range. The Chi Squared test was used to establish bivariate associations for each potential predictor and JEY utilization. To ascertain association between different predictor variables and outcomes, a multivariate Poisson Regression with robust confidence intervals was used to generate prevalence ratios (PR) with 95% confidence intervals. The first multivariate model studied the association of these predictors with use of the JEY. The second multivariate analysis with similar predictor variables was performed for the outcome transport delay. As the study included several explanatory variables that could be correlated to each other, an assessment of collinearity was performed to check variance inflation factors (VIFs) as a post-estimation test.

## Results

### Sample Characteristics of Women and Facilities

A total of 1,126 women delivered in the facilities over the recruitment period; 1,005 women were enrolled in the study, 121 were excluded. Some of these women resided outside the study district (n = 102) or had missing information (n = 5), and 14 refused to participate. Participant characteristics are shown in Table 2. The median age of mothers was 23 years, 78% were from rural areas, and 52% lived below the poverty line. Women delivered in 81 different facilities, 63 public and 18 private. All private and nine public facilities provided EmOC services i.e. performed the six signal functions. Among the public facilities, only 5% were primary or community health centers.

**Table 2:** Characteristics of Study Sample.

	Total	%
**Individual Characteristics**	n = 1005	%
Primi-Parous	385	38
No/Primary Education	540	54
Caste		
* SC*	246	24
* OBC*	461	46
* ST*	139	14
* General*	159	16
BPL Card	527	52
Rural Residence	780	78
ASHA Accompanied	411	41
Previous knowledge of JEY	803	80
Referred Mother	111	11
**Distance**		
Distance travelled		
* 0–10 km*	550	55
* 10–20 km*	269	27
* ≥20 km*	186	18
**Place of delivery**		
Delivered in a **Public** facility providing EmOC	453	50
Delivered in a **Public** facility not proving EmOC	461	50
Delivered in a **Private** facility providing EmOC	91	100

Of the 1,005 women in the study, 914 delivered in public facilities and hence were JSY beneficiaries. More than half (n = 544) of the women delivered in a facility that provided EmOC, 83% of these were in a public facility.

### Modes of Transportation to the Health Facilities

The various modes of transportation used by the women are shown in Table 3. The overall uptake of JEY transport was 35%. However utilization differed between the districts; 24%, 46%, 52% respectively. Among the 353 women who used JEY transport, 97% delivered in a public facility. The median distance travelled by JEY users was the longest of all single modes of transportation (11.3**km). The median time reported between deciding to leave their home and arriving at the facility for JEY users was greater (120 minutes) compared to 60 minutes for mothers with their own transportation and 75 minutes for mothers who hired a vehicle. It was similar to those women who used public transportation (Table 3).

**Table 3:** Mode of transport, median for time, cost and distance travelled (interquartile range) for all mothers (n = 1005).

Transport	Frequency (%)	Time (minutes)*	Cost (Rs.)	Distance (km)
**Hired Vehicle**	383 (38)	75 (60–150)	200 (70–500)	7.0 (2.6–16.2)
**Janani Express**	353 (35)	120 (60–210)	0 (0–0)	11.3 (6.4–18.8)
**Own Vehicle**	135 (13)	60 (30–120)	50 (0–100)	4.1 (1.6–11.4)
**Other** †	65 (6)	60 (30–120)	0 (0–0)	2.1 (1.0–5.3)
**Public Transport**	43 (4)	120 (60–180)	40 (20–100)	7.42 (3.7–19.9)
**Multiple mode**	26 (3)	180 (120–300)	150 (30–300)	20.4 (9.4–28.2)

*The time taken from deciding to leave home and arriving at the facility.

† other modes of transport includes walking and borrowing a vehicle.

### Characteristics of Janani Express Yojana Users and Non-users

Characteristics of users and non-users are shown in Table 4. The JEY usage was greater among women from a lower socioeconomic position; 40% of women with low educational status, 44% of all rural women and 55% of all women from scheduled tribes utilized the JEY. However, 48% (n = 255) of all women living below the poverty line paid for their transport. Ninety-six percent (n = 215) of urban women were non-JEY users and 74% of these women paid for their transportation.

**Table 4:** Description of JEY users, the Bivariate and Multivariate Poisson Regression model with prevalence ratios (PR) n = 1005. Column % are presented.

	JEY User	JEY Non-User	Bivariate	Multivariate
	n = 353 (%)	n = 652 (%)	PR (95% CI)	
No/Primary Education	218 (62)	322 (49)	**1.4 (1.2–1.7)**	1.1 (0.9–1.3)
Secondary & Higher	135 (38)	330 (51)	*Reference*	
BPL Card	192 (54)	335 (51)	1.1 (0.9–1.3)	**-**
No BPL Card	161 (46)	317 (49)	*Reference*	
Caste				
* SC*	91 (26)	155 (24)	**1.6 (1.2–2.2)**	1.3 (0.9–1.7)
* OBC*	148 (42)	313 (48)	**1.4 (1.0–1.9)**	1.1 (0.9–1.5)
* ST*	77 (22)	62 (10)	**2.4 (1.7–3.3)**	**1.6 (1.2–2.2)**
* General*	37 (10)	122 (19)	*Reference*	
Rural Residence	343 (97)	437 (67)	**9.9 (5.4–18.2)**	**4.5 (2.4–8.5)**
Urban Residence	10 (3)	215 (33)	*Reference*	
Distance travelled				
* 0–10 km*	154 (44)	396 (61)	*Reference*	
* 10–20 km*	124 (35)	145 (22)	**1.7 (1.4–2.0)**	1.1 (0.9–1.4)
* ≥20 km*	75 (21)	111 (17)	**1.4 (1.2–1.8)**	1.1 (0.9–1.4)
ASHA Accompanied				
* No*	92 (26)	502 (77)	*Reference*	
* Yes*	261 (74)	150 (23)	**4.4 (3.5–5.4)**	**3.1 (2.5–3.8)**

A majority (61%) of the women who used JEY transport delivered in a non-EmOC public facility, a small subset (7.7%) of women delivered on the way to the health facility. While only 3% of users delivered in a private facility, 12.4% of non-JEY mothers delivered in a private facility.

### Predictors of Service Use

Three quarters of women who used the JEY transport reported that the ASHA arranged the transport to the health facility, 90.2% of these mothers were accompanied to the facility by the ASHA. The ASHA did not arrange transport for 91% (n = 592) of the non-users. In the multivariate model, women whom the ASHA accompanied to the facility were 3 times more likely to use JEY (PR = 3.1; 95% CI, 2.5–3.8). Rural women were 4.5 times more likely to travel by JEY (PR = 4.5; 95% CI, 2.4–8.5). Women belonging to scheduled tribes retained significance, all other socioeconomic characteristics lost significance in the model (Table 4).

### Factors and Reasons Related to Transport Related Delays

Thirty-one percent (n = 303) of women experienced more than a two hour delay from deciding to leave their home and arriving in the facility. As presented in Table 5, transportation related delays represented 68.6% (n = 208) of all delays and were more prevalent among JEY users (29.8%) than non-users (16.7%). Women who experienced a transport related delay reported a median time of four hours to reach the facility after deciding to leave. In the multivariate model (Table 5), JEY users were as likely to have delays as women who used private hired vehicles, public transportation or multiple modes of transport.

**Table 5:** The proportion of women experiencing a transport delay by characteristic & multivariate Poisson regression (n = 972*) Column % are presented.

	Delayed	Not Delayed	Bivariate	Multivariate
**Place of Residence**	n (%)	n (%)	PR (95% CI)	
Urban	13 (6)	197 (26)	*Reference*	
Rural	195 (94)	567 (74)	**4.1 (2.4–7.1)**	**2.9 (1.6–5.2)**
**Transport**				
* Hired*	70 (34)	305 (40)	*Reference*	
* Own/borrowed*	10 (5)	138 (18)	**0.4 (0.2–0.7)**	**0.4 (0.2–0.7)**
* JEY*	104 (50)	248 (32)	**1.6 (1.2–2.1)**	1.2 (0.9–1.6)
* Other* †	24 (11)	73 (10)	1.3 (0.9–2.0)	1.2 (0.9–1.8)
**Distance**				
* 0–10 km*	80 (38)	448 (59)	*Reference*	
* >10–20 km*	70 (34)	193 (25)	**1.8 (1.3–2.3)**	1.3 (0.9–1.8)
* ≥20 km*	58 (28)	123 (16)	**2.1 (1.6–2.8)**	**1.6 (1.2–2.2)**
**ASHA Accompanied to Facility**				
No	103 (50)	461 (60)	*Reference*	
Yes	105 (50)	303 (40)	**1.4 (1.1–1.8)**	0.9 (0.7–1.2)

*33 women did not have all the time points necessary to calculate the delay.

† Public Transport, walking and multiple modes of transport.

JEY users reported waiting for JEY to arrive (70%) as the most common reason for a transport related delay while non-users reported arranging their own transport (48%), lack of attendant to accompany them to the hospital (18%), poor roads or weather conditions (13%) or waiting for JEY to arrive (13%) as the main causes of delay.

## Discussion

This is the first study on Janani Express Yojana (JEY), a unique public private partnership (PPP) for emergency obstetric transportation in central India. This study addresses utilization, equity in use and timeliness of the service. It throws light on some of the strengths and weaknesses of this innovative initiative that supports the JSY cash transfer program to promote institutional delivery.

The JEY service model complements the cash transfer program by providing vulnerable women at risk of maternal morbidity/mortality transport to a health facility to give birth. JEY uptake was greater in women with lower socioeconomic status. A study of the distribution of use suggests the intervention was utilized by significant proportions of the most vulnerable population subgroups, which need the service most, promoting equity in access. However, the overall uptake of the service was relatively low; half of all BPL women still paid for alternative transportation to a facility. Therefore, there is a need for additional research to understand why more women are not utilizing the free service.

The service, being free of charge to the user, does save money for the women who utilize it; however 30% of all users experience transport related delays which are comparable to those experienced by women traveling by public transportation. These delays are likely due to a combination of long waiting times, poor road conditions, the remoteness of the areas serviced and a possible shortfall in the number of vehicles available, in relation to the demand for the service. Strong monitoring mechanisms by the state are required to ensure a high quality service.

### Using Public Private Partnership Service Models for Emergency Transport

Transportation difficulties in gaining access to facility based care are well known and documented for low and middle income countries with high maternal mortality rates. These include high cost of transport, challenging diverse geographical terrain, poor referral communication processes, and suboptimal distribution and location of health facilities [17], [18]. In addition to operating in the context of poor infrastructures, arrangements for emergency transport have often been informal and ‘ad-hoc’ in the absence of reliable emergency transportation facilities provided by the public sector. The operational cost and maintenance of an ambulance fleet is expensive so in the unlikely event it is even available, the service is often too expensive for poor families to use [3].

High political priority was given to increasing institutional deliveries through the JSY cash transfer program in Madhya Pradesh. The following conditions created the opportunity for a public private partnership (PPP). Madhya Pradesh is a predominately rural state with high levels of poverty, which make it difficult for people to afford additional transport costs. In addition there is no state-run transport service, but there is a strong heterogeneous network of private sector transport companies. In this context, a PPP makes financial and practical sense; it saves the government resources that would otherwise be needed to own and maintain its fleet of emergency obstetric transport vehicles, while still ensuring a service free-of-cost to the user. Strong stewardship from the state is essential to ensure women receive the service required and to deter the introduction of corruption and inefficient processes that could accompany third party management. To the authors’ knowledge, JEY is the first public private partnership set up solely for emergency obstetric transport in India.

### Opportunity to Expand JEY Services

Despite high awareness of JEY throughout the community, there were women who did not use the service and could have benefited from it. More than half the women in the study had to pay for their transport, the majority of them living below the poverty line. Only 13% of all mothers had immediate access to their own transportation. There is an opportunity for the JEY to expand its services to more women in need, especially among the women living in rural areas where free transportation alternatives are not readily available. Our study found the role of the ASHA in arranging transport and accompanying the women to the facility to be essential in the uptake of JEY. More engagement with the ASHA at the community level could possibly improve the service uptake.

### Equitable Access to JEY Transport Service for Rural Poor Women

Geographical distance may play a small role in determining access to facility based delivery care for women living in urban areas with an established functioning health care infrastructure. However, in rural areas, where road infrastructures are weak, transportation alternatives are few and functional health facilities are further, distance can present a serious barrier to accessing adequate delivery services thus perpetuating high maternal mortality [18]–[20]. Babinard et al [21] reiterate the important role transport plays in facilitating efficient and effective care by enabling access to care, especially for rural areas. In Madhya Pradesh, thick forest vegetation covers one third of the area and the other two thirds comprises hills, plains, plateaus, and rivers. The diverse terrain, which varies from flat plains to forest to large hills, presents a unique challenge for the development of an effective road network system and subsequently development of an emergency obstetrics transportation system [22]. In our study, we found the highest uptake took place among the more rural districts (2&3) and nearly 50% of all rural women utilized the service. JEY decreases the geographical barrier among poor, illiterate, rural women and promotes equitable geographical access.

### JEY Delaying Access to EmOC Care?

Skilled birth attendance alone will not prevent maternity mortality. A study in Gujarat, India [23] found between 42% and 52% of maternal deaths occurred at home or in transit to the facility; long travel times and delays in obtaining transport no doubt contribute to persistent high maternal mortality. In our study, it took more than 4 hours for 50% of the women who traveled by JEY to arrive at a facility *after* they decided to leave their home. Given the unpredictable nature of complications during pregnancy and childbirth and the association between in-hospital maternal mortality and more than 120 minutes to reach the facility, these women are at a greater risk of dying in the event of an unforeseen complication.

Women who utilized the JEY transport took the same amount of time to reach the facility as women who traveled by public transportation and were just as likely to experience a delay. JEY use or waiting for a JEY vehicle accounted for more than half (53%) of the delays. This delay is likely to have converted some potential users into non-users. While rural areas were predominantly serviced by the JEY, the median distance traveled (11.5 km) by these women is unlikely to account for such a long journey time. Though not studied in our study, the lengthy waiting times could be indicative of an incompetent management system or insufficient number of JEY vehicles in operation for the respective demand. Ensuring timely services are delivered requires the government to implement better monitoring mechanisms to improve time efficiency than currently available.

### Methodological Considerations

This study only includes women who delivered in a health facility. The nature of access barriers among women who delivered at home may differ from those identified in this study. The uptake of the service within the community is still unknown. However, the proportion of home deliveries is low; a recent study showed home deliveries only accounted for 5% of all deliveries in one of our study districts (unpublished data). Nevertheless, the study is limited by being only facility based.

The delay variable was created based on maternal reports of time. This variable could be affected by the mother’s ability to accurately recall the events preceding the delivery. Recall bias was minimized by administering the questionnaire shortly after delivery and family members present during the interview were also consulted to verify the time sequence of events. The delay variable does not differentiate between the amount of time spent waiting for the vehicle to arrive and actual time spent in transport. Since JEY is servicing more rural areas, it is difficult to distinguish whether the delay is caused by actual distance or other unknown variables.

In our sample, the proportion of women living below the poverty line was reflective of the general population in districts 2 and 3. However, the proportion of women living below the poverty line in district 1 was much higher in our sample than the general population. The majority of the sample was recruited from public facilities; therefore poor women in the JSY program may have been over sampled. Since more rural poor women tend to use the service, this may have inflated the overall estimated uptake of the program in district 1.

Women’s intent to use JEY and the reasons for the specific modes of transportation employed were not explored in this study. Insight into the decision making process of the women and their family members pertaining to the mode of transportation could help explain the low uptake of the service.

Poisson regression with robust confidence intervals was selected for the analysis due to the outcome (JEY user) being a common event (>30%). In this setting the odds ratios generated by a logistic regression would differ greatly from the relative risk and lead to an inflated effect [24]. The prevalence ratio however provides a reliable point estimate of the size of the relative effect, based on the ratio between the outcome and predictor variables leading to a more accurate reflection of the relative risk [25]. The robust confidence intervals account for under-dispersion, in which the data is more dispersed than predicted by the model [26].

## Conclusions

The JEY is India’s first emergency transportation model dedicated exclusively to transporting mothers giving birth. This innovative service has been well utilized particularly by mothers in rural areas. The utilization of vulnerable women, uneducated and tribal, signifies success in promoting equitable access to care. This service definitely fulfills an existing gap, however further operations research to inform efforts to (i) improve coverage as there are still many poor rural women who are paying for their transport and (ii) improve time efficiency by minimizing the transport related delays for pregnant women will be useful.

## Supporting Information

Appendix S1 **Detailed information on the selection of health facilities.**
(JPG)
