# Supplementary figures and images for: Utilization of a State Run Public Private Emergency Transportation Service Exclusively for Childbirth: The Janani (Maternal) Express Program in Madhya Pradesh, India
Source: PLoS One. 2014 May 14;9(5):e96287. doi: 10.1371/journal.pone.0096287 (PMC4020755; doi:10.1371/journal.pone.0096287)

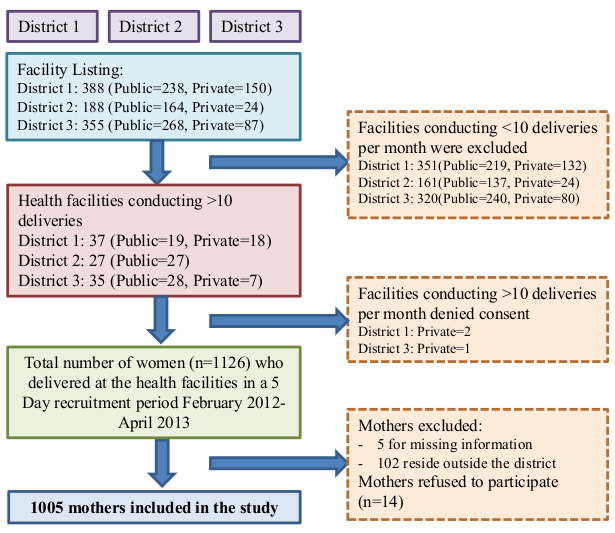

Supplement: Appendix S1 — Detailed information on the selection of health facilities. (JPG) [file pone.0096287.s001.jpg]
